# Relationship between Pain, Physical Activity, Screen Time and Age among Young Children during the COVID-19 Pandemic

**DOI:** 10.3390/healthcare12161635

**Published:** 2024-08-16

**Authors:** Reem M. Basuodan, Afnan Gmmash, Mshari Alghadier, Reem A. Albesher

**Affiliations:** 1 Department of Rehabilitation Science, Princess Nourah bint Abdulrahman University, Riyadh 11671, Saudi Arabia; rmbasoudan@pnu.edu.sa; 2 Department of Physical Therapy, King Abdulaziz University, Jeddah 21589, Saudi Arabia; asgmmsh@kau.edu.sa; 3 Department of Physical Therapy and Rehabilitation, Prince Sattam bin Abdulaziz University, Alkharj 16278, Saudi Arabia; m.alghadier@psau.edu.sa

**Keywords:** physical activity, screen time, children, COVID-19 pandemic

## Abstract

Introduction: During the COVID-19 pandemic, many countries implemented restrictions, social distancing measures, and lockdowns to limit the spread of the disease. These lockdowns have affected children’s screen time (ST), pain, and physical activity (PA) levels. The present study aimed to explore the relationships between ST, pain, age, and PA before, during, and after the COVID-19 pandemic. Methods: The caregivers of 329 children (median age = 8 years) filled out an online self-reported survey about the children’s PA, ST, and pain before, during, and after the COVID-19 curfew. Spearman’s rank correlation coefficient was used to explore the associations between these variables. Results: After the curfew, pain that existed before the pandemic had a weak negative relationship with PA intensity (−0.11, *p* = 0.04) and a weak positive relationship with ST (r = +0.12, *p* = 0.04). There was a strong positive relationship between ST in all time periods (*p* > 0.01). PA and ST had a weak negative relationship (*p* > 0.05) during the curfew and after the curfew but not before the COVID-19 pandemic. Age had a weak positive correlation with ST in all time periods (*p* > 0.01). In addition, ST was affected by the curfew. Conclusion: The study findings indicated that young children had longer ST during the curfew and after the curfew compared with before the curfew. Increasing PA could lessen children’s ST, which could, in turn, increase the probability that their general pain would decrease.

## 1. Introduction

Managing pain in children and adults is crucial for maintaining a high quality of life and enabling active engagement in essential daily activities [1,2]. In children, pain can be attributed to multiple factors, such as trauma, psychological stress, and acute or chronic illnesses [3]. Several types of pain affect children such as headaches, abdominal pain, musculoskeletal pain, and multiple pains [4,5]. Musculoskeletal pain, including lower back pain, has been shown to be higher in inactive school-aged children and adolescents compared with their active peers [6]. In children, pain can be exacerbated by engaging in sedentary activities, such as passive screen time (ST) and online games [7]. During the COVID-19 pandemic, the physical activities (PAs) of children, adolescents, and adults decreased significantly. The disrupted daily routine and decreased levels of PAs affected children’s health [8]. Children’s diets were also affected [9]. Consequently, rates of obesity, musculoskeletal issues, and other health-related conditions have been rising since the pandemic [10]. Meanwhile, PA did not rapidly bounce back to normal levels after the pandemic [11].

After the spread of COVID-19, some countries imposed curfews or lockdowns, in which recreational activities and stores were closed and walking around on the streets without a permit was prohibited. Similarly, in the kingdom of Saudi Arabia, a partial curfew (from 6am–7pm) that later became a complete 24 h lockdown was imposed all over the country. During the lockdown, leaving the house was not allowed without a permit for an urgent reason such as a hospital visit [12]. These daily curfews were placed as a preventative measure to control the spread of COVID-19 [13]. The curfews restricted children’s participation in routine daily activities, which could have contributed to their pain levels. Children’s age and sedentary behaviors, including long periods of ST, might have also played a role in increasing their pain levels. The prevalence of pain and the use of digital technology among children increased with the dramatic lifestyle changes caused by the pandemic-related lockdowns. A large number of adolescents as well as adults maintained this minimal level of activity after the pandemic [14,15,16].

Along with children’s physical health, their mental health was also affected by the pandemic. Children, especially females, were more susceptible to anxiety, family issues, and depression during the pandemic [17,18]. The psychological impact of the pandemic and the isolation it caused led to issues associated with inadequate sleep quality and migraines [16]. Oversleeping and sleep disturbances were further affected by low income and greater quarantine time [19]. Children were not prepared and trained to be resilient against such a traumatic global disaster. In addition, families’ mental health and dynamics influenced children’s wellbeing. Disturbed sleeping schedules aggravated children’s psychological stress. There was a documented rise in the diagnosis of mood disorders and the use of related prescription medication during this tough time [20,21]. The pandemic imposed some necessary adaptations to combat the spread of the virus. One of these adaptations was switching to distant learning, which has also affected children’s physical and mental health. Online schooling automatically increased children’s ST. Available online resources exist as preventative measures to increase students’ physical activity [15]. Some of the preventive measures recommended by the World Health Organization include staying hydrated, walking, and engaging in simple physical activities [22]. Children aged 4–17 are encouraged to engage in moderate-to-vigorous exercise for at least 60 min daily to maintain a healthy lifestyle [23]. However, school-aged children may not have received an adequate level of advocacy regarding the importance of the recommended daily activity as their PA levels were largely affected during the pandemic [24].

More evidence is warranted to reveal the effect of the pandemic on children before, during, and after the pandemic to assist stakeholders in reversing any adverse effects and prevent them from occurring in case another containment policy is enforced for any similar reason in the future. The present study aimed to understand the relationships among children’s pain, PA levels, age, and ST before the COVID-19 pandemic, during the COVID-19-related curfews, and after the COVID-19 curfews. This study also aimed to identify factors that could contribute to pain in young children so that these factors could be addressed by researchers and policymakers in the future.

## 2. Materials and Methods

### 2.1. Study Sample

This cross-sectional study, which included an online survey administered via social media, was conducted during the official school summer holiday in 2022, when there was a partial curfew in place in the country due to the COVID-19 pandemic. The population selected for sampling comprised caregivers of six- to nine-year-old children in Saudi Arabia. Participants were recruited through various channels. The survey link was distributed via social media channels such as Instagram, Twitter, and WhatsApp groups, which mothers or caregivers would visit frequently. Restrictions, including partial curfew, social distancing, and restricted movement, meant an online self-reported survey was the only distribution channel available. Additionally, the survey link was posted via email to all Princess Nourah bint Abdulrahman University in Riyadh (PNU) users.

Ethics approval for the study was obtained from the ethics committee of PNU in Riyadh, Saudi Arabia (H-01-R-059), in accordance with the Helsinki Declaration on ethical standards in human research. Informed consent was obtained from all caregivers prior to commencing the data collection process.

### 2.2. Instrumentation

A self-reported survey was designed to collect information about three dependent variables, pain, PA, and ST, during three time periods, before the COVID-19 pandemic, during the COVID-19 pandemic-related curfew, and during the COVID-19 pandemic but after the curfew ended (i.e., when social distancing was in effect but there was no curfew). The survey had three main parts. Part one concerned demographic information, including the age and gender of the child. Part two included a question about pain frequency in which the respondents were asked to use a five-point Likert scale to describe how often the child suffered from pain during the COVID-19 pandemic (no pain, rarely, sometimes, often, always). In addition, the respondents were asked to use a four-point Likert scale to describe changes related to pain that the child first experienced before the COVID-19 pandemic (no pain, better/pain decreased, no change, worse/pain increased).

Part three provided clear definitions and adopted questions [25] for the dependent variables PA and ST (Appendix A). It also included questions about the child’s PA frequency in days (e.g., How many days did your child practice athletic activities for at least 60 min?), PA intensity in days (e.g., How many days did your child practice athletic activities to strengthen his/her bones and muscles, such as jumping rope, climbing high objects, running, dancing, or performing aerobic exercise with a family member?), and PA duration in minutes (e.g., For approximately how many minutes per day did your child practice athletic activities to strengthen his/her bones and muscles, such as jumping rope, climbing high objects, running, dancing, or performing aerobic exercise with a family member?). These questions were repeated for each time period under study. Valid and reliable PA questions were adopted from Prochaska et al. [25]. Part three also included three questions about ST adopted from Melkevik et al. [26] about the total number of hours that the child spent watching screens, engaging in screen play, and using devices generally.

All questions were written in English and then translated into Arabic in a three-step process by a certified translation center to ensure the validity of the Arabic version of the survey. To confirm the clarity of the questions in Arabic, eight caregivers from different educational levels and backgrounds reviewed and commented on the survey twice before the study commenced. All comments were considered, and modifications were made to the survey.

### 2.3. Data and Statistical Analysis

All continuous variables were screened to assess normality using Kolmogrov–Smirnov tests and graphical inspection methods, including histograms, normal quantile–quantile (Q-Q) plots, and box plots. Since the variables did not follow a normal distribution, nonparametric analysis was used. Regarding descriptive statistics, frequencies, percentages, and medians were calculated for all variables. Spearman’s rank correlation coefficient was used to explore the associations for the continuous and categorical variables. The level of significance was set at *p* ≤ 0.05 at 95% confidence intervals (CIs).

## 3. Results

### 3.1. Descriptive Statistics

A total of 329 surveys completed by caregivers of six- to nine-year-old children were included in the study. The median age of the children was 8 years. There were slightly more female children (54%) than male children (47%) in the sample. The descriptive statistics of the children’s demographic characteristics and the variables examined across the three periods under study are presented in Table 1 and Table 2.

### 3.2. Correlation

Pain that started before the COVID-19 pandemic had a significant but weak inverse association with PA intensity (r = −0.11, *p* = 0.04) and a significant but weak positive association with ST after the curfew (r = +0.12, *p* = 0.04). No significant associations were found between pain behavior and the variables for the other two time periods (i.e., before the COVID-19 pandemic and during the curfew). Similarly, no significant associations were found in terms of pain frequency during any of the time periods.

In all the time periods (after the curfew, during the curfew, and before the COVID-19 pandemic), ST had a significant and strong positive relationship (r = +0.82, +0.62, and +0.58, respectively; *p* = 0.00). In the last seven days, ST had significant but weak inverse correlations with PA frequency, intensity, and duration during the curfew (r = −0.12, −0.19, and −0.25, respectively; *p* = 0.03, 0.00, and 0.00, respectively). It also had significant but weak inverse correlations with PA intensity and duration after the curfew (r = −0.23 and −0.25, respectively; *p* = 0.00 and 0.00, respectively). ST had significant but weak inverse correlations with the PA variables during the curfew (r = −0.11, −0.11, and −0.18, respectively; *p* = 0.05, 0.04, and 0.00, respectively) and during the last seven days (r = −0.13 and −0.14, respectively; *p* = 0.02 and 0.00, respectively). However, for the period before the COVID-19 pandemic, the correlations between ST and all the other variables were weak, negative, and nonsignificant (Table 3).

Age had a significant but weak positive correlation with ST during all three time periods (r = 0.18, +0.15, and +0.17, respectively; *p* = 0.00, 0.01, and 0.00, respectively). No significant associations were found between age and the PA variables during any of the time periods (Table 3).

## 4. Discussion

This study aimed to investigate the association between pain, PA, and ST during three time periods during the COVID-19 pandemic. Our study showed that the pain that started before COVID-19 became worse in children who had longer STs. However, the pain decreased in those who were involved in higher PA levels after the curfew. Also, PA generally correlated inversely with ST during and after the curfew but not in the period before the COVID-19 pandemic. Children spent double the time on screens during and after COVID-19 than they did before COVID-19. The ST spent by children was proportional in all periods. Interestingly, children’s age was correlated positively with ST in all periods.

Children’s mental health deteriorated during and after the pandemic [27]. This decline in their psychological health could have caused pain that started during COVID-19 [28,29]. Our study showed that there was a weak correlation between pain and ST after the curfew in the period of COVID-19. Pain could have been higher in children with high levels of anxiety, psychological distress, and sleep disturbances [30]. This is consistent with what was found in older school-aged children, as pain was also associated with ST [31]. Pain was also significantly associated with the intensity of PA. Pain limits participation in physical activity. According to the global recommendation for PA, children should participate daily in at least moderate levels of physical activities to maintain a healthy lifestyle [32]. Staying active can have numerous positive effects on children’s overall health, but the lockdown decreased children’s ability to stay physically active [33]. This study did not focus on the relationship between specific types of pain, ST, and PA, which could explain the insignificant and weak correlations. Future studies should focus on exploring the type of pain associated with prolonged ST. 

Our study showed that ST increased during and after the curfew that was imposed during the pandemic. This increase was expected with the lockdown, especially for young children with the limited access to outdoor activities and social distancing. Switching to distance learning may have also contributed to the elevation in the number of hours spent on screens, which was also evident in high school students [34]. This is consistent with the results of a study carried out in Saudi Arabia [35]. Previously documented challenges related to distance learning include dependent learning and increased ST without parental supervision. Supporting parents during online learning can be accomplished by advising children to be physically active during classes and assisting them in writing meaningful PA-related goals to encourage offline PA [36,37].

Evenson et al. (2023) [35] found that the amount of sedentary behavior increased with the application of COVID-19 restrictions. Our study also showed that ST before and during COVID-19 increased while children’s PA intensity and duration decreased. An inverse relationship between ST and PA was previously reported in the literature [38]. Increased ST and limited in-person communications could negatively impact children’s overall health [39]. ST could also negatively influence children’s academic attainment [40]. This relationship should be further explored to understand how these two variables are correlated. One study found that ST may limit the favorable impact of healthy physical fitness habits in children. Thus, the exact intensity and duration of PA and children’s fitness levels should be measured to be able to report the precise influence of ST on children’s health [40].

Regular at-home PA may assist in limiting the negative squeal of sedentary behaviors. Parents and caregivers, as well as teachers, should encourage children to participate in different types of activities that are interesting to these children to decrease their passive engagement in ST activities [41,42,43,44].

Age was a significant factor that affected the amount of time spent on screens. An increase in ST with age could be because older children spend more time on social and recreational activities [45]. Children’s engagement in ST could also be affected by the amount of time their parents spend on screens [46]. Parents are advised to limit their online activities and connect with their children. Children should be encouraged to explore nature and engage in outdoor activities to promote emotional and behavioral wellbeing [47]. 

### Limitations and Future Studies 

Although this study recruited a large number of participants from different regions in Saudi Arabia, the cross-sectional design of this study could confine the generalizability of the results. The pain questions used in this survey were not thoroughly validated. However, content and face validity were established for these questions. More studies should be conducted to investigate the relationship between different types of pain and ST before and after the pandemic. The results of this study cannot confirm a causative relationship between the studied variables. Experimental studies are needed to identify the best methods that can be used to overcome unavoidable sedentary behaviors that could accompany any pandemic-related lockdowns. Accessibility to virtual reality games and activities could be used by children as an alternative to improve their engagement in physical activities if limitations to outdoor activities were imposed [48].

## 5. Conclusions

During the pandemic, the amount of ST increased with age before, during, and after the curfew. The result of this study shows that the pandemic-related curfew increased children’s ST, which in turn could affect children’s participation in physical activities. In case of another pandemic imposing curfews, efforts should be directed toward limiting children’s ST and incorporating actions that aim at increasing their engagement in activities to reach the recommended guidelines for physical activity.

## Figures and Tables

**Table 1:** Descriptive statistics of the children’s demographic characteristics and the variables.

Variables		Count from a Total of 329	%	Median
Age	6 y	67	20%	8 y
	7 y	78	24%	
	8 y	96	29%	
	9 y	92	28%	
Screen hours	After curfew	-	-	8 h
	During curfew	-	-	8 h
	Before COVID-19	-	-	4 h
Gender	female	178	54%	
	male	155	47%	
Pain frequency	0 (No pain)	293	89%	0
	1 (Rarely)	11	3%	
	2 (Sometimes)	23	7%	
	3 (Often)	6	2%	
	4 (Always)	0	0%	
Pain behavior	0 (No pain)	306	93%	0
	1 (Better/pain decreased)	3	1%	
	2 (No change)	15	5%	
	3 (Worse/pain increased)	9	3%	
Pain type	Headache	4	11%	
	Abdominal	6	17%	
	Musculoskeletal	19	56%	
	Fever-associated body ache	5	15%	
	Other	6	11%	

Y, years; h, hours; %, percentage.

**Table 2:** Children’s PA frequency, intensity, and duration (n = 329).

N of Days	After Curfew N (%)	During Curfew N (%)	Before COVID-19 N (%)		After Curfew N (%)	During Curfew N (%)	Before COVID-19 N (%)		After Curfew N (%)	During Curfew N (%)	Before COVID-19 N (%)
PA frequency					PA intensity			PA duration			
0 d	114 (35%)	49 (15%)	57 (17%)	0 d	130 (40%)	112 (34%)	65 (20%)	0 min	113 (34%)	92 (28%)	57 (17%)
1 d	60 (18%)	43 (13%)	40 (12%)	1 d	51 (16%)	48 (15%)	41 (12%)	≥30 min	127 (39%)	143 (43%)	116 (35%)
2 d	62 (19%)	43 (13%)	73 (22%)	2 d	57 (17%)	48 (15%)	87 (26%)	<30 min	54 (16%)	63 (19%)	85 (26%)
3 d	30 (9%)	49 (15%)	64 (19%)	3 d	39 (12%)	52 (16%)	60 (18%)	≥60 min	39 (12%)	35 (11%)	75 (23%)
4 d	27 (8%)	23 (7%)	46 (14%)	4 d	15 (5%)	27 (8%)	22 (7%)		-	-	-
5 d	15 (5%)	17 (5%)	25 (8%)	5 d	11 (3%)	15 (5%)	24 (7%)		-	-	-
6 d	7 (2%)	6 (2%)	10 (3%)	6 d	8 (2%)	7 (2%)	11 (3%)		-	-	-
7 d	7 (2%)	32 (10%)	18 (5%)	7 d	22 (7%)	24 (7%)	23 (7%)		-	-	-

N, numbers; %, percentage.

**Table 3:** Variables and correlation coefficient analysis during the three time periods.

Variables	Age	PA Frequency	PA Intensity	PA Duration	Screen Time	PA Frequency	PA Intensity	PA Duration	Screen Time	PA Frequency	PA Intensity	PA Duration	Screen Time
		After curfew, r (*p*)				During curfew, r (*p*)				Before COVID-19, r (*p*)			
Pain frequency	+0.08 (0.16)	+0.03 (0.58)	−0.10 (0.08)	0.00 (0.95)	+0.07 (0.18)	+0.02 (0.74)	−0.10 (0.07)	+0.02 (0.76)	+0.07 (0.19)	+0.03 (0.60)	+0.02 (0.78)	+0.10 (0.08)	+0.04 (0.52)
Pain behavior before COVID-19	+0.03 (0.64)	+0.06 (0.31)	−0.11 (0.04 *)	−0.03 (0.53)	+0.12 (0.04 *)	+0.02 (0.66)	−0.09 (0.11)	−0.01 (0.88)	+0.08 (0.16)	+0.01 (0.88)	+0.02 (0.72)	+0.06 (0.29)	+0.04 (0.49)
Age		+0.01 (0.83)	−0.02 (0.78)	−0.05 (0.33)	-	+0.05 (0.41)	+0.05 (0.34)	+0.04 (0.49)	-	+0.04 (0.45)	+0.03 (0.65)	+0.05 (0.34)	-
Screen time in last 7 days	+0.18 (0.00 *)	−0.10 (0.08)	−0.23 (0.00 *)	−0.25 (0.00 *)	-	−0.12 (0.03 *)	−0.19 (0.00 *)	−0.25 (0.00 *)	+0.58 (0.00 *)	+0.02 (0.73)	−0.09 (0.09)	−0.00 (0.93)	-
Screen time during curfew	+0.15 (0.01 *)	−0.04 (0.48)	−0.13 (0.02 *)	−0.14 (0.00 *)	+0.82 (0.00 *)	−0.11 (0.05 *)	−0.11 (0.04 *)	−0.18 (0.00 *)	-	+0.05 (0.42)	+0.01 (0.89)	+0.08 (0.16)	-
Screen time before COVID-19	+0.17 (0.00 *)	−0.01 0.79	−0.01 (0.82)	−0.03 (0.58)	+0.65 (0.00 *)	−0.07 (0.21)	−0.06 (0.30)	−0.10 (0.06)	-	−0.07 (0.20)	−0.05 (0.33)	−0.07 (0.17)	-

PA, physical activity; r, Spearman’s rank correlation coefficient; *p*, significance; *, *p* ≤ 0.05.

## Data Availability

Data are contained within the article.

## Appendix A. Physical Activity and Screen Time Adopted Questions

**Moderate-to-Hard Physical Activity** Physical Activity means any activity that increases your heart rate, cut your breath and gasps for some time. Physical activity can be performed by playing sports, school activities, playing with friends or walking to school. Some examples of physical activity are: running, biking, dancing, skateboarding, swimming, football, water skiing or snowboarding. For the following question, please summarize all the times you were physically active every day. For the past seven days, how many days have you been physically active for at least 60 min per day? Answer options: 0 days, 1 day, 2 days, 3 days, 4 days, 5 days, 6 days, 7 days **Screen-Based behaviors** How many hours do you spend a day, in your free time, watching TV, videos (including YouTube or similar services), DVDs and other on-screen entertainment? How many hours do you spend a day, in your free time, playing computer, tablet (e.g., iPad), smartphone or other electronic devices (not including motion games or fitness)? How many hours do you spend every day, in your free time, using electronic devices such as computers, tablets and (e.g., iPads) or smartphones for other purposes such as: performing homework, email messaging, using social networks such as Twitter and Facebook, chatting and surfing the internet? The following nine response options cover the previous three questions: None at all. About 30 min a day. About an hour a day. About Two hours a day. About Three hours a day. About Four hours a day. About Five hours a day. About Six hours a day. About Seven hours a day.
